# Evaluating the Efficacy of Combined Intralesional Sodium Stibogluconate Plus Topical Imiquimod on Healing and Risk of Scarring of Cutaneous Leishmaniasis: A Case-Control Study

**DOI:** 10.7759/cureus.23200

**Published:** 2022-03-15

**Authors:** Samer A Dhaher, Thaar A Hussein

**Affiliations:** 1 Dermatology, College of Medicine, University of Basrah, Basrah, IRQ; 2 Dermatology, Basrah Teaching Hospital, Basrah, IRQ

**Keywords:** topical, scarring, imiquimod, sodium stibogluconate, cutaneous leishmaniasis

## Abstract

Background: A combination treatment of cutaneous leishmaniasis (CL) that hastens the healing and reduces the chance of scarring, especially in aesthetically receptive sites, is required.

Objectives: To evaluate if a combination of intralesional sodium stibogluconate (SSG) injection and topical imiquimod 5% cream (IMI) accelerates healing and improves the quality of scars from CL.

Patients and Methods: A prospective, placebo-controlled, randomized clinical trial was conducted at Basrah Teaching Hospital, Basrah, southern Iraq from 2017 to 2019 on a cohort of patients with CL. Eligible patients were injected intralesionally with sodium stibogluconate (SSG) weekly for six weeks and randomized to receive either topical imiquimod 5% cream (IMI group) or topical emollient cream (placebo group). The healing rate and scar quality were assessed at week six.

Results: One hundred twenty-one patients completed the trial (66 [55%] males, mean age SD: 34.1 years). The clinical healing rate was significantly higher in the IMI group than in the placebo group (94% versus 74%, p <0.05). A high rate of scars was noticed in both groups (66.6% in the IMI group and 91.2% in the placebo group). However, superficial non-atrophic scars were more frequent in the IMI group (40% versus 26%), while deep atrophic scars were more evident in the placebo group than in the IMI group (65.2% versus 26.6%, p<0.05).

Conclusions: Combined intralesional SSG plus topical imiquimod was beneficial in accelerating CL healing and improving scar quality, and should be considered when CL is located in aesthetically sensitive areas.

## Introduction

Leishmaniasis is a broad spectrum of chronic infections in humans caused by species of Leishmania (flagellated protozoans). Transmission is via the bite of infected female sandflies [1]. Cutaneous leishmaniasis (CL) is endemic in many countries around the world, including Asia and, to a lesser extent, in the Mediterranean basin. It is estimated that about 90% of CL occurs in the Middle East, Brazil, and Peru [2]. The clinical presentation of CL primarily depends on the host’s cell-mediated response and on the species of Leishmania involved and usually starts as small, well-circumscribed solitary or multiple papules at the inoculation site, slowly enlarging over several weeks into ulcerated or verrucous nodules or plaques on exposed sites such as the face, neck, arms, and legs. The diagnosis can be confirmed by detection of the presence of amastigotes in dermal macrophages within skin biopsy stained with Wright, Giemsa, or Feulgen stains [3]. Recently, the polymerase chain reaction (PCR)-based method has become the most specific and sensitive diagnostic test [4]. Without treatment, CL typically resolves within six to 15 months, but with disfiguring lifelong scars. Local infiltration or systemic administration of pentavalent antimony drugs (sodium stibogluconate and meglumine antimoniate) is the mainstay treatment option. However, such treatment has many limitations, such as cost, duration, drug resistance, and potential toxicity [5]. Imiquimod is an immunomodulator drug that stimulates innate and adaptive immune pathways, resulting in antiviral, antitumor, and immunoregulatory properties [6]. Imiquimod increased the production of cytokines (IFN-γ, IL-6, and TNF-α), induced natural killer (NK) cell activity, and released nitric oxide from macrophages and an agonist for the toll-like receptor 7 (TLR7) on macrophages and dendritic cells leads to a Th1-type immune response [7,8]. To date, there is no standard and universally accepted treatment option dedicated to CL and the choice is primarily based on the geographic location and availability of the drug [9]. Meanwhile, we assume that any given treatment should aim not only to induce healing but also preclude the possibility of disfiguring scars, and a combined treatment that targets the killing of the parasite by multiple mechanisms will result in a better response than a single treatment. Currently, the residual scarring that follows the resolution of active CL is considered an "inactive" form of CL, and as 50% of the lesions are located on the face, scars may trigger social stigma and impair quality of life [10]. In Iraq, a hyperendemic area for disease activity, the scar of CL is termed "Ukhut", which means "sister", as it remained for life and profoundly affects the mental and social health of the patients. Therefore, it is essential for any treatment option to not only speed up healing but also reduce the risk of scarring, especially for lesions located in cosmetically sensitive sites [11]. The current study aims to test whether the combination of topical Imiquimod 5% cream plus intralesional sodium stibogluconate (SSG) injection is beneficial in accelerating healing, increasing the cure rate, and minimizing the risk of the scar.

## Materials and methods

We enrolled patients with CL in a randomized, placebo-controlled trial to compare the clinical effects of adding topical imiquimod cream versus topical emollient cream to intralesional SSG injection. The study was approved by the Ethical Committee of the College of Medicine, University of Basrah (Approval No: 03040831-2017).

Eligibility for inclusion in the trial includes all patients with CL who were referred to our hospital and fulfilled the following criteria: identification of the amastigote stage of Leishmania in each lesion using Giemsa stain on histopathological examination, above the age of 18 years, and cooperative patients who agreed to participate in the study and signed the formal consent. Patients with mucosal lesions, pregnancy, co-existing chronic systemic diseases like liver failure, renal failure, or diabetes mellitus, previous allergic reaction to SSG or imiquimod, and lesions larger than 5 cm or more than five in number were excluded.

The study took place from January 2017 to November 2019 at Basra Teaching Hospital, Department of Dermatology and Venereology, Basra, Iraq, which is an endemic area for CL. Cases were referred from primary health care centers, which provide health services to more than 2 million people.

Eligible patients were randomly assigned to two groups: IMI and placebo. The IMI group received intralesional SSG (100mg/ml) injections without local anesthesia weekly for six weeks using a 30-gauge needle. The entire size of the lesion was infiltrated with a sufficient amount of medication (0.5 ml for each 1 cm) until blanching took place, plus topical imiquimod 5% cream containing 250 mg was applied by the patient at night every other day for six weeks as a thin layer on the entire lesion(s), including 0.5 cm margin of normal skin, and rubbed gently until vanished. The Placebo group received an intralesional injection of SSG in a way similar to the first group, plus topical emollient cream was provided to the patients in a small container containing 20 grams of petrolatum and was applied every other day once at night and labeled as the control group.

The treatment primary endpoint was the number and percentage of patients who achieved clinical cure with or without scar after week six. Clinical cure was defined as complete healing by re-epithelialization with flattening of the individual lesion, while post-healing scars were evaluated as a secondary endpoint, and classified into either superficial non-atrophic scars or deep atrophic scars. The progress throughout the study was assessed by measurement of the following parameters: surface area, degree of erythema, and induration at baseline and then weekly for six weeks. The lesion surface area was measured after taking a photograph at a fixed distance and calibration with a camera of 12.2 Megapexiel, then the photographs were analyzed using UTHSCSA (University of Texas Health Science Center at San Antonio) image tool version 3.00 (a software program used to mark the erythema of a leishmaniasis lesion and measure its surface area in cm^2^). The degree of erythema was assessed and graded according to its intensity as follows: full erythema (dusky red), moderate erythema (liliaceous erythema), mild erythema, and no erythema (normal skin color). The induration was assessed by palpating the lesion and comparing it with the adjacent normal skin and was scored as follows: fully indurated (full resistance to pinching), moderately indurated (moderate resistance to pinching), mildly indurated (mild resistance to pinching), and not indurated (no resistance to pinching like normal skin).

Participants were randomly assigned to both groups using a computer-generated randomization list. To ensure a balance in sample size across both groups, a list of block randomization with a block size of four was prepared by an independent statistician. For the assignment of all participants, a random sequence of blocks was predetermined and sent to the pharmacist who dispensed either topical imiquimod cream or placebo containers. The researchers (SD and TA) responsible for enrollment, assignment of participants, outcome assessment, and data analysis were blinded by the topical drugs prescribed.

To detect a significant difference in the cure rate of 50% in the placebo group and at least 80% in the imiquimod group with a two-sided 5% significant level and a power of 80%, 53 patients would be required per group. Assuming that a dropout rate of 10% is anticipated, 58 patients will be needed for each group. Data analysis was performed using IBM SPSS version 25 (IBM Corp., Armonk, NY, USA). Mean (SD) was used to report descriptive data and frequencies and percentages for quantitative data. An intention-to-treat analysis was performed at the end of the trial (at week six). For determination of the statistically significant difference between an independent variable in both groups, the chi-square test of association was used. p-values less than 0.05 were considered significant. 

## Results

**Figure 1:**
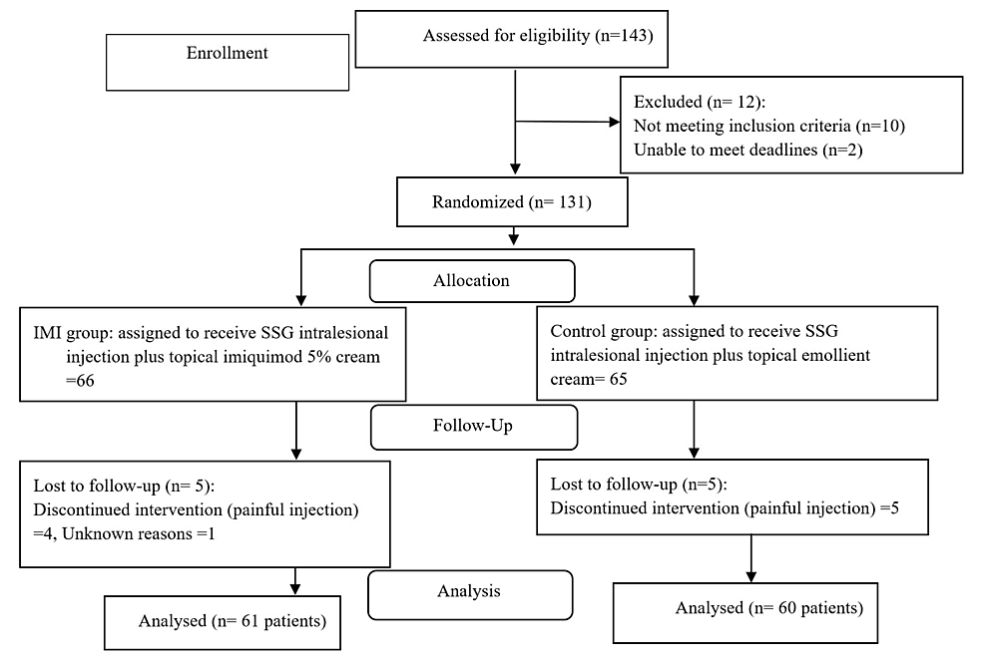
Flow chart of the study participants. SSG: sodium stibogluconate, IMI: imiquimod

The flow chart of the study population is demonstrated in Figure 1, and the patients' demographic characteristics are shown in Table 1.

**Table 1:** The demographic features of study participants.

Variable	No and percentage
Total no. of patients	121
Gender	males: 66(54.8%)
	females: 55(43.8%)
Mean age ±SD	34.8±10.5 years
Mean duration ±SD	2.5±1.8 months
Type of the lesion	Wet 43(35.5%)
	Dry 78 (64.4%)
Site of lesions	Head and neck: 8(6.3%)
	Upper limb: 69 (57.1%)
	Lower limb: 44(36.5%)

In the IMI group, 57 (94%) and 44 (74%) patients in the placebo group were healed after six weeks and the difference was statistically significant (p<0.05), while 19 (33.3%) in the IMI group and four (8.7%) patients in the placebo group were healed without a scar and the difference was statistically significant (p<0.05%). In contrast, healing with deep atrophic scars was statistically significantly higher in the placebo group than in the IMI group (65.2% versus 26.6%, p<0.05) (Table 2).

**Table 2:** The cure rate and type of scar in both groups after six weeks of treatment. IMI: imiquimod

Group (No)	Non healed No (%)	Healed No (%)	Healed without scar No (%)	Healed with scar No (%)	Deep atrophic scar No (%)	Superficial non-atrophic scar No (%)
IMI Group (61 patients)	4 (6%)	57 (94%)	19(33.3%)	38 (66.6%)	15 (26.6%)	23(40%)
Placebo Group (60 patients)	16(26%)	44 (74%)	4(8.7%)	40(91.2%)	29(65.2%)	11(26%)
P-value	0.043		0.047			

**Figure 2:**
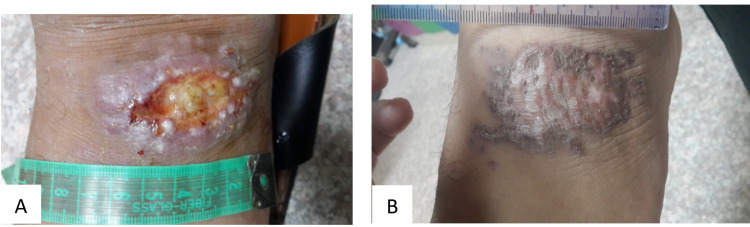
(A) cutaneous leishmaniasis at baseline, (B) the same lesion after six weeks of treatment with sodium stibogluconate injection plus topical imiquimod shows healing with a superficial non-atrophic scar and post-inflammatory hyperpigmentation.

**Figure 3:**
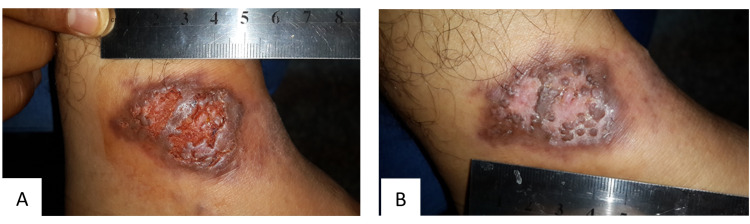
(A) Cutaneous leishmaniasis before treatment with intralesional sodium stibogluconate plus topical emollient cream. (B) The same lesion after six weeks shows healing with remarkably deep atrophic scarring.

Figures 2 demonstrate the type of scar in the IMI-treated group, while Figure 3 shows the healing with a deep atrophic scar in the placebo group.

In the IMI group, there was a significant reduction in surface area at week six of treatment compared to the baseline value (p<0.05), while the placebo group did not show a significant reduction in surface area during the period of treatment (p > 0.05) (Table 3).

**Table 3:** The effect of treatments in both groups on the surface area of cutaneous leishmaniasis (mean SD/cm2) and percentage of reduction each week for six weeks. IMI: imiquimod

Group	Mean (SD) of the surface area of the lesions /cm^2^						
	Baseline	1week	2week	3week	4week	5week	6week
IMI (61 patients)	6.2(5)	5.6(4.8)	5.2(4.8)	4.7(4)	4.2(4)	3.9(3.9)	3.6(3.9)
Percentage of reduction		12.4	21.8	31.44	39.98	48.4	55.45
P-value		0.59	0.0338	0.177	0.073	0.036	0.019
Placebo (60 patients)	5.4(3.9)	5.4(3.8)	5.31(3.9)	5.22(3)	5.31(3)	5.02(4)	4.97(3)
Percentage of reduction		1.83	4.01	6.58	8.75	11.92	13.16
P-value		0.957	0.882	0.805	0.744	0.658	0.626

At baseline in both groups, there was no significant difference in the degree of erythema (p>0.05). However, there was a significant reduction in the percentage degree of erythema after the first week of starting treatment. At the end of the sixth week, 51 (84%) patients of the IMI group and 10 (16%) patients of the placebo group showed no erythema and the difference was statistically significant (p value<0.05) (Table 4). 

**Table 4:** The degree and percentage of erythema in both groups at baseline and each week for six weeks. IMI: imiquimod

Visit	Treatment group	No erythema No (%)	Mild erythema No (%)	Moderate erythema No (%)	Severe erythema No (%)	P-value
Baseline	IMI	0 (0%)	0 (0%)	15 (25%)	46 (75%)	0.763
	Placebo	0 (0%)	0 (0%)	11 (19%)	49 (81%)	
1^st^ week	IMI	0 (0%)	6 (9%)	30 (50%)	25 (41%)	0.015
	Placebo	0 (0%)	0 (0%)	16 (26%)	44 (74%)	
2^nd^ week	IMI	0 (0%)	13 (22%)	46 (75%)	2 (3%) 21 (35%)	0.000
	Placebo	0 (0%)	0 (0%)	39 (65%)		
3^rd^ week	IMI	4 (7%)	41 (68%)	16 (25%)	0 (0%)	0.000
	Placebo	0 (0%)	11 (19%)	42 (70%)	7 (11%)	
4^th^ week	IMI	21 (35%)	40 (65%)	0 (0%)	0 (0%)	0.000
	Placebo	0 (0%)	31 (52%)	29 (48%)	0 (0%)	
5^th^ week	IMI	48 (78%)	13 (22%)	0 (0%)	0 (0%)	0.000
	Placebo	0 (0%)	44 (74%)	16 (26%)	0 (0%)	
6^th^ week	IMI	51 (84%)	10 (16%)	0 (0%)	0 (0%)	0.000
	Placebo	10 (16%)	42 (70%)	8 (14%)	0 (0%)	

At baseline in both groups, there was no significant difference in the degree of induration, while after the first week of treatment, there was a significant reduction in the degree of induration in the IMI group compared to the placebo group (80% versus 46%, p<0.05). At the end of treatment, 78% of the IMI group and 25% of the placebo group showed no induration and the difference was statistically significant, (p value<0.05) (Table 5).

**Table 5:** The degree and percentage of erythema in both groups at baseline and each week for six weeks. IMI: imiquimod

Visit	Treatment group	No erythema No (%)	Mild erythema No (%)	Moderate erythema No (%)	Severe erythema No (%)	P-value
Baseline	IMI	0 (0%)	0 (0%)	15 (25%)	46 (75%)	0.763
	Placebo	0 (0%)	0 (0%)	11 (19%)	49 (81%)	
1^st^ week	IMI	0 (0%)	6 (9%)	30 (50%)	25 (41%)	0.015
	Placebo	0 (0%)	0 (0%)	16 (26%)	44 (74%)	
2^nd^ week	IMI	0 (0%)	13 (22%)	46 (75%)	2 (3%) 21 (35%)	0.000
	Placebo	0 (0%)	0 (0%)	39 (65%)		
3^rd^ week	IMI	4 (7%)	41 (68%)	16 (25%)	0 (0%)	0.000
	Placebo	0 (0%)	11 (19%)	42 (70%)	7 (11%)	
4^th^ week	IMI	21 (35%)	40 (65%)	0 (0%)	0 (0%)	0.000
	Placebo	0 (0%)	31 (52%)	29 (48%)	0 (0%)	
5^th^ week	IMI	48 (78%)	13 (22%)	0 (0%)	0 (0%)	0.000
	Placebo	0 (0%)	44 (74%)	16 (26%)	0 (0%)	
6^th^ week	IMI	51 (84%)	10 (16%)	0 (0%)	0 (0%)	0.000
	Placebo	10 (16%)	42 (70%)	8 (14%)	0 (0%)	

## Discussion

In recent years, cutaneous leishmaniasis (CL) has reached hyperendemic levels in conflict zones such as Syria, Iraq, and Afghanistan [12]. From a patient perspective, it is important to recognize that the burden of disease does not end when the active lesion is resolved, but rather carries on with the residual scar left behind after healing, which is lifelong and hard to remove cosmetically.

With the role of infected humans as reservoirs, the relatively long course of the disease, and the high probability of leaving scarring in the area with physical and psychological consequences for the patient, it is recommended to treat cutaneous leishmaniasis early [13]. As long as the lesions persist locally and the parasite does not migrate, it is reasonable to treat these infections intralesionally [14]. Intralesional or systemic SSG is considered the benchmark treatment for CL as it induces a high clinical cure rate ranging from 72% to 94% [15,-17]. Even though CL scarring was indeed a foregone conclusion, it hasn't been included in the criteria used to evaluate treatment response. The consideration of the use of imiquimod 5% cream in our study is based on the fact that the activation of toll-like receptor 7 by imiquimod could work in at least two ways to enhance the resolution of Leishmania infections. Firstly, it could directly stimulate macrophages to synthesize nitric oxide, resulting in the direct killing of the parasite [18]. Secondly, imiquimod could mediate a better anti-Leishmania Th1 immune response that would result in the production of IFN-γ and macrophage activation resulting in enhanced parasite killing [19]. Imiquimod also has direct effects on scar formation, as it has recently been shown that topical application of imiquimod 5% cream can reduce keloid formation and scarring after surgical excision [20]. This was explained by the local overproduction of interferons following topical imiquimod application which had an antifibrotic effect by inhibiting both college and glycosaminoglycan production and promoting collagenase activity leading to a clinical decrease in the size of the keloid. 

In the current study, we found that treating CL with SSG plus imiquimod resulted in a significantly higher cure rate than SSG with emollient cream. Imiquimod-treated patients had a higher rate of healing without scarring, while those healed with non-atrophic superficial scars had a higher imiquimod treatment rate than those with deep atrophic scars. These findings support the assumption that the addition of imiquimod 5% cream has an advantageous effect. Not only does it speed up and increase the cure rate, but it also reduces the chance of scar formation, making these scars more cosmetically acceptable, which would be especially important when CL is situated in an aesthetically sensitive area such as the face or neck.

The application of imiquimod cream as a treatment for CL has been evaluated in previous studies with controversial results. Seeberger treated 12 patients with imiquimod cream using a standard protocol (topical application three times a week), and a further three patients served as the control group. The lesions were regressed but not healed within the first two to four weeks in 10 of the 12 patients, whereas in two patients no change was observed and they concluded that imiquimod as monotherapy was ineffective in the treatment of CL [21]. A similar finding was also reported by Firooz et al. in a placebo-controlled study using topical imiquimod or placebo cream combined with meglumine antimoniate intramuscular injection and they noticed that at the end of the four-week treatment period, a clinical cure was similar in both groups (44.1% vs 48.3%). They showed no beneficial effect of combining imiquimod with meglumine antimoniate [22]. On the other hand, Arevalo et al. in an open-label prospective study re-treated 12 patients who had previously not responded to systemic meglumine antimoniate with a combined topical imiquimod three times weekly and intramuscular pentavalent antimony. They reported that the cure rate was 90% [23]. Similarly, in a randomized controlled trial, Miranda-Verastegui et al. treated 40 patients with CL with meglumine antimoniate (20 mg/kg) intramuscular injection daily, plus either topical imiquimod 5% cream or vehicle that was applied every other day for 20 days. After three months, the cure rate in the imiquimod-treated group was 72% versus 35% in the other group [18]. In Crawford et al. in an open-label trial, 99 patients with cutaneous leishmaniasis were allocated to three treatment groups. They received combined intralesional meglumine antimoniate plus imiquimod, imiquimod alone, or intralesional meglumine antimoniate alone for 40 days. The authors reported 37%, 35%, and 23% response rates, respectively, where a better response was observed in patients who received either imiquimod alone or in combination with meglumine antimoniate compared with patients treated with meglumine antimoniate alone [24]. The differences in outcomes between these studies are most likely due to differences in study design, sample size, and administration route, as well as the potential for Leishmania species to develop treatment resistance, which has been observed in some places of the world, such as Pero and Iran [25]. While a poor cure rate has been linked to the use of imiquimod alone, it is likely that when paired with an antimonial drug, imiquimod has a synergistic effect, and improves the cure rate through immunologically enhanced parasite elimination. Even more, SSG administration routes also have an impact on the healing process, as intralesional administration of SSG, although painful, is easy to administer in the outpatient setting with a low risk of systemic toxicity, and will also ensure a high concentration of medication at the site of the lesion, which will result in a better response than intramuscular administration.

The study had some limitations. First, the study relied on evidence from a single-center trial with limited external validity, so the generalizability of the intervention needs to be tested on a larger population in a multi-centric study. The second was drug costs, although the local health authorities subsidized SSG and disbursed it at no cost to those with CL.

## Conclusions

Our findings support the notion that combining intralesional sodium stibogluconate weekly injection with topical imiquimod applied on alternative days over a six-week course was more effective than sodium stibogluconate plus topical emollient for the treatment of cutaneous leishmaniasis. Such a combination has the advantage of not only boosting the cure rates but also improving the quality and reducing the risk of scarring of cutaneous leishmaniasis and should be considered for those patients who have their lesions on aesthetic sites such as the face and neck.
